# Towards the development of *Bacillus subtilis *as a cell factory for membrane proteins and protein complexes

**DOI:** 10.1186/1475-2859-7-10

**Published:** 2008-04-04

**Authors:** Jessica C Zweers, Imrich Barák, Dörte Becher, Arnold JM Driessen, Michael Hecker, Vesa P Kontinen, Manfred J Saller, L'udmila Vavrová, Jan Maarten van Dijl

**Affiliations:** 1 Department of Medical Microbiology, University Medical Center Groningen and University of Groningen, Hanzeplein 1, P.O. Box 30001, 9700 RB Groningen, The Netherlands; 2 Institute of Molecular Biology, Slovak Academy of Sciences, Dubravska cesta 21, 84554 Bratislava 45, Slovak Republic; 3 Institute for Microbiology, F.L. Jahnstr. 15, D17487 Greifswald, Germany; 4 Department of Molecular Microbiology, Groningen Biomolecular Sciences and Biotechnology Institute, University of Groningen, Kerklaan 30, 9751 NN Haren, The Netherlands; 5 Infection Pathogenesis Laboratory, Department of Viral Diseases and Immunology, National Public Health Institute, Mannerheimintie 166, FIN-00300 Helsinki, Finland

## Abstract

**Background:**

The Gram-positive bacterium *Bacillus subtilis *is an important producer of high quality industrial enzymes and a few eukaryotic proteins. Most of these proteins are secreted into the growth medium, but successful examples of cytoplasmic protein production are also known. Therefore, one may anticipate that the high protein production potential of *B. subtilis *can be exploited for protein complexes and membrane proteins to facilitate their functional and structural analysis. The high quality of proteins produced with *B. subtilis *results from the action of cellular quality control systems that efficiently remove misfolded or incompletely synthesized proteins. Paradoxically, cellular quality control systems also represent bottlenecks for the production of various heterologous proteins at significant concentrations.

**Conclusion:**

While inactivation of quality control systems has the potential to improve protein production yields, this could be achieved at the expense of product quality. Mechanisms underlying degradation of secretory proteins are nowadays well understood and often controllable. It will therefore be a major challenge for future research to identify and modulate quality control systems of *B. subtilis *that limit the production of high quality protein complexes and membrane proteins, and to enhance those systems that facilitate assembly of these proteins.

## 1. Introduction

### 1.1 History

**Figure 1:**
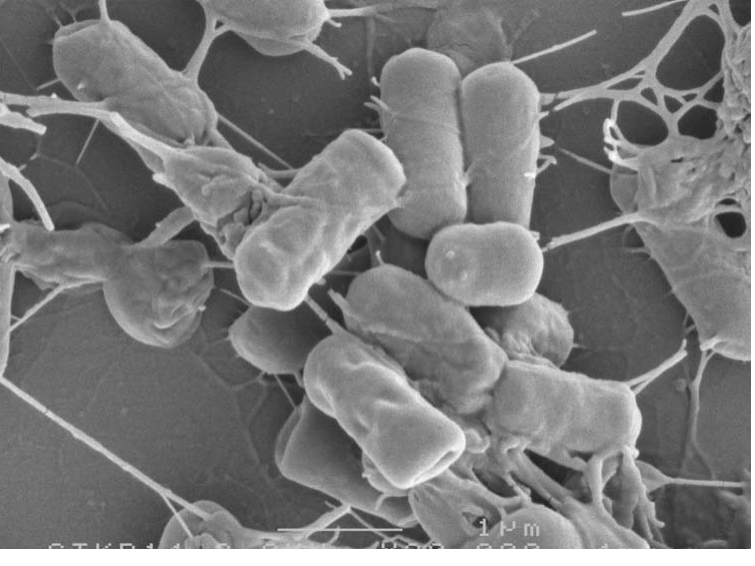
Scanning electron microscopic image of *B. subtilis *168.

*Bacillus subtilis *is a sporulating rod-shaped Gram-positive bacterium (Fig. 1), which thrives in the soil. Like most of its closest relatives *B. subtilis *is non-pathogenic, and *B. subtilis *has even been awarded GRAS (Generally Recognized As Safe) status by the US Food and Drug Administration. The first known application of *B. subtilis *dates back more than a thousand years, when it was already used to produce natto, a Japanese food product consisting of fermented soybeans. Nowadays, *B. subtilis *is best known as a source of useful enzymes and fine biochemicals, and as an attractive host for the production of heterologous proteins. Many different enzymes, like proteases and amylases, originating from *B. subtilis *and related *Bacillus *species are being used in industry for a wide range of different applications [1-6]. Importantly, *B. subtilis *is able to produce and secrete large quantities of proteins into the culture medium. Therefore, this organism is widely regarded as a prolific "cell factory" for industrial enzymes and biopharmaceuticals [1,7].

*B. subtilis *is genetically highly amenable as it develops genetic competence for DNA binding and uptake. This is one of the prime reasons why bacilli have been extensively used in both applied and fundamental scientific research for more than 50 years. In 1990 a European-Japanese research collaboration was started, with the aim to sequence the entire genome of *B. subtilis *strain 168. This has led to the publication of the entire annotated genome sequence in 1997 [8]. A subsequent international project has led to the identification of all essential genes in *B. subtilis *[9]. Today, *B. subtilis *is one of the best understood of all living organisms, and it has become the paradigm for research on Gram-positive bacteria. Detailed data sets on the transcriptome [10,11], proteome [12], secretome [13] and metabolome [14] of *B. subtilis *are available, representing a rich source of information for research on *Bacillus *species. Importantly, the relatively close relationships between *B. subtilis *and clinically relevant Gram-positive pathogens also make this organism highly relevant for research on potential targets for novel antimicrobials and anti-infectives.

### 1.2 *Bacillus subtilis *as a host for protein production

Currently the most commonly used bacterial host for industrial production of heterologous proteins is *Escherichia coli*. Advantages of its use as a production host for proteins are that it can be grown easily in large fermentations, and that it is genetically amenable and able to produce large quantities of proteins. However, in *E. coli *the produced proteins usually accumulate within the cells where they have a high potential to aggregate, resulting in the formation of inclusion bodies. To acquire the protein, the inclusion bodies need to be separated from the cell and the proteins subsequently need to be recovered from the inclusion bodies. Moreover, the outer membrane of *E. coli*, and of Gram-negative bacteria in general, contains lipopolysaccharide (LPS or endotoxin), which is highly pyrogenic and needs to be totally removed before the produced proteins can be used for clinical purposes.

*B. subtilis *has excellent fermentation capacities that are equal to, if not better, than those of *E. coli*. In addition, *B. subtilis *is also capable of producing large quantities of proteins. However, in contrast to *E. coli, B. subtilis *lacks an outer membrane and is able to secrete proteins directly into the medium. Therefore, the secreted proteins can be purified easily from the medium in their active form, which simplifies the downstream processing considerably. Although most of the proteins that are commercially produced by *B. subtilis *are secreted into the medium, there are also successful examples of cytoplasmic protein production in *B. subtilis *[15].

Like all living organisms, *B. subtilis *has cellular quality control systems. These facilitate the production of high quality proteins, and remove misfolded and incompletely synthesized proteins [16,17]. Unfortunately, cellular quality control systems also represent significant bottlenecks in heterologous protein production [18,19]. This poses a fascinating challenge for cell factory engineering since inactivation of quality control systems can improve protein production yields, but these improved yields might be at the expense of product quality. Clearly, a reduced product quality would be an unwanted effect, especially if the product is a biopharmaceutical. Notably, the mechanisms underlying the degradation of secretory proteins in *B. subtilis *are nowadays fairly well-understood and, in many cases, the yields of "fragile" secretory proteins could be improved significantly by engineering of the cellular machinery for protein quality control [20].

Successful strategies for engineering of *B. subtilis *to improve protein production include the knockout of extracellular and/or intracellular proteases [21-23], overexpression of chaperones and folding catalysts [24-27], overexpression of components of the secretion machinery, and/or modification of the cell wall microenvironment [28,29]. Besides engineering the host, also the expression system used to produce the protein can be modified in order to improve production and/or secretion, for example by the use of strong or inducible promoters [30-32]. Another strategy is to modify the protein that is being produced itself, for example by selecting an optimal signal peptide [33,34], or by rendering the protein less sensitive for degradation through site-specific mutagenesis [35]. The latter protein modification approach has the disadvantage that it can affect the functionality and folding of the protein.

Over all, it has become clear that a wide range of approaches for modification of *B. subtilis *can be applied to further improve this important cell factory for production of cytoplasmic and secretory proteins. Nevertheless, there are still many proteins that remain recalcitrant to such approaches. These include membrane proteins and proteins that are part of cytoplasmic or membrane-associated protein complexes.

### 1.3 The membrane proteome as a resource for biomedical and biotechnologicaly relevant proteins

To maintain the cellular homeostasis, the cytoplasmic membranes of bacteria are largely impermeable to ions, the majority of nutrients and signaling molecules. The vital communication between the cytoplasm, transmembrane compartments and the extracellular milieu is facilitated through membrane-embedded proteins. They typically account for about 30% of open reading frames in prokaryotic and eukaryotic genomes [36], and they carry out a diverse range of functions in vital processes such as cellular growth and division, maintaining cell integrity, energy transduction, signal sensing and transduction, cell-cell interactions, and transmembrane transport processes (Table 1). Membrane proteins are without any doubt the most important group of proteins in terms of current drug targets. Despite their functional and biotechnological importance, the study of membrane proteins has remained difficult due to their hydrophobicity. Accordingly, they generally require detergents to remain soluble upon extraction from the membrane. The presence of detergents, however, complicates the biochemical and structural analysis of membrane proteins. Consequently, high-resolution structural data is available for only very few membrane proteins, while X-ray crystal structures are available for daily increasing numbers of soluble proteins. To date, only the most abundant membrane proteins have been characterized in some detail.

**Table 1:** Overview of determined and predicted functions of membrane proteins in *B. subtilis*. The numbers of membrane proteins belonging to each functional category are shown.

**Cell envelope and cellular processes**		**522**
	cell wall	40
	transport/binding proteins and lipoproteins	305
	signal transduction (sensors)	30
	membrane bioenergetics	35
	motility and chemotaxis	20
	protein secretion	15
	cell division	8
	sporulation	47
	germination	13
	transformation/competence	9
**Intermediary metabolism**		**62**

	Metabolism of carbohydrates	17
	Metabolism of amino acids	12
	Metabolism of nucleotides and nucleic acids	7
	metabolism of lipids	12
	metabolism of coenzymes and prosthetic groups	9
	metabolism of phosphate	4
	metabolism of sulfur	1
**Information pathways**		**21**

	DNA restriction/modification and repair	2
	Transcription regulation	6
	ribosomal proteins	1
	protein modification	4
	Protein folding	8
**Other functions**		**60**

**Unknown**		**490**

**Figure 2:**
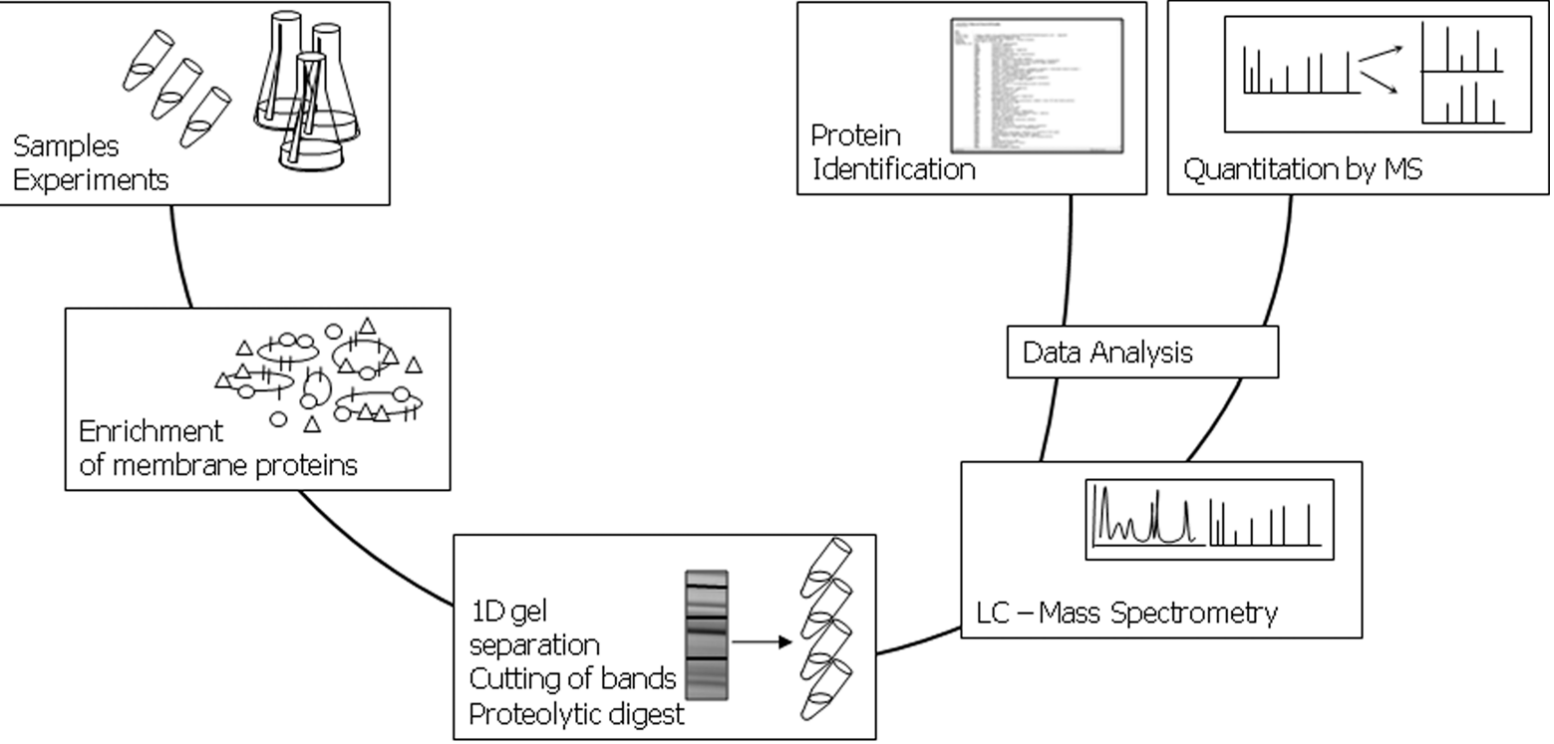
**Workflow for the analysis of membrane proteins via 1D gel LC-MS/MS. **The current workflow for analysis of the *B. subtilis *membrane proteome involves several steps. First bacteria are cultivated under appropriate conditions and samples are withdrawn. Next, the harvested cells are disrupted, and the membrane fraction is enriched in successive centrifugation steps. Membrane proteins are separated by one-dimensional SDS PAGE, excised from the gel, and digested with an appropriate protease. The peptides thus obtained are analyzed by liquid chromatography and mass spectrometry for protein identification. Different approaches can be employed for quantitative membrane proteomics. For *B. subtilis*, these have so far involved stable istotope labeling with amino acids (SILAC) such as ^13^C6-^15^N2 lysine, and ^14^N/^15^N metabolic labeling.

Not only the analysis of the properties of individual membrane proteins is difficult, but the same applies even more so to complexes of membrane proteins as well as the entire membrane proteome. Thus, the analysis of membrane proteomes, in general, has so far been relatively unproductive compared to analyses of cytosolic proteomes, cell wall proteomes and exoproteomes. This also applies to the *B. subtilis *membrane proteome [13,37,38]. First studies to investigate the *B. subtilis *membrane proteome were undertaken by Bunai et al. [39] and by Dreisbach et al. [37]. To this end, different methods for membrane protein solubilization were combined with gel-based, semi-gel-based and gel-free proteomics techniques (Fig. 2). More than 700 proteins were identified in the *B. subtilis *membrane; 122 of these proteins contain predicted N-terminal signal peptide-like sequences that may serve in membrane targeting. From the membrane proteins that were identified by Eymann *et al*., 268 proteins contain at least one potential membrane spanning domain [37], and 134 contained four or more potential transmembrane domains. Notably, most detected membrane proteins of *B. subtilis *are still of unknown function and this is in fact also true for a multitude of membrane proteins in other species. The functionally defined proteins are permeases and transporters, dehydrogenases, subunits of respiratory complexes, oxidoreductases, ATP-synthase components, two-component signal transduction proteins, penicillin-binding proteins, signal peptidases and proteins involved in cell motility, cell division, autolysis, chemotaxis, and osmoregulation.

Several of the identified *B. subtilis *membrane proteins have potential biotechnological applications. For example, cytochrome P450-like proteins can be exploited for the bioconversion of a wide range of substrates [40]. Other identified *B. subtilis *membrane proteins, especially the essential ones, may represent potential new targets for the development of novel antimicrobial or anti-infective drugs.

### 1.4 Production of membrane proteins

Due to their roles in key cellular processes, membrane proteins represent interesting subjects for fundamental scientific research on their structure-function relationships. In addition, these proteins are crucially important from a pharmaceutical perspective, because they are drug targets that are relatively easy to address being exposed on the extracytoplasmic side of the membrane. Thus, pharmaceuticals acting on these proteins do not always need to enter the cell. Unfortunately, most membrane proteins are naturally present in relatively low amounts in the cells. This makes it difficult to obtain such proteins in sufficient amounts for functional and structural analyses. Moreover, achieving high-level expression of membrane proteins has turned out to be very difficult. Several high-throughput screens for membrane protein overproduction have been performed, most of them using *E. coli *as the expression host [41-43]. In some of these studies a clear correlation was found between the success of expression and the number of predicted transmembrane helices [44,45]. However, in contradiction with these results, a screen on overexpression of *E. coli *inner membrane proteins in *E. coli *indicated that there is no clear correlation between the ability to overproduce a membrane protein and protein size, the number of transmembrane helices or specific sequence characteristics [46]. Remarkably, in yet another screen for expression of 49 *E. coli *membrane proteins it was even found that the majority of successfully expressed proteins had a high number of transmembrane helices [47]. Furthermore, it was proposed that not only properties of the protein itself would determine whether a particular membrane protein could be overproduced successfully. Other important parameters were the *E. coli *strain used for overproduction, the type of detection/purification tag fused to the overproduced protein, and N-terminal or C-terminal fusion of such a tag to the overproduced protein [47]. Another problem is that in most cases the produced proteins mainly accumulate in the cytoplasm and aggregate. In fact, there are only very few examples where it was shown that a large amount of the protein was inserted correctly in the membrane [41,45,48]. It is thought that the presence of high amounts of proteins in the membrane can affect the integrity of the membrane and thereby have a toxic effect on the cells. However, although cytoplasmic accumulation can prevent this toxicity, the purification of overproduced membrane proteins from the cytoplasm is not preferred for most applications, since the protein may either be folded incorrectly, inactive, or both. Notably, the overproduced membrane proteins can be hard to purify and the proteins can be readily lost during purification or subsequent crystallization for structural analyses. Therefore, there is a clear and generally recognized need for systems to overproduce correctly membrane-inserted membrane proteins in large amounts.

To date, it is unknown to what extent *B. subtilis *is exploitable for high-level membrane protein production. However, membrane protein biogenesis usually requires the same general secretion (Sec) pathway that is used by bacteria to direct the vast majority of exported proteins to extracytoplasmic cellular locations or the growth medium (reviews: [49,50]). This seems also to be true for *B. subtilis *[13,38]. Since the Sec pathway of *B. subtilis *has a huge capacity for protein secretion, often to commercially significant gram per liter levels, there is presently no reason to assume that the *B. subtilis *Sec pathway will be less effective in inserting proteins into the cytoplasmic membrane. Thus, it can be anticipated that *B. subtilis *will turn out a highly suitable host for membrane protein production at high levels.

### 1.5 Protein complexes and the interactome

For many years, *B. subtilis *has been a widely appreciated model organism in studies on basic cellular processes, such as cell division, DNA replication, and cell differentiation. Thus, this organism was a logical choice for subsequent functional genomics, transcriptomic and proteomics research on these cellular processes. As the next step in reaching a more global molecular understanding of cellular processes, new proteomics and systems biological methodologies are currently being explored for analyzing post-translational modifications, protein stability/degradation and protein interaction networks [51].

The proteome of any living organism is divided into structured protein interaction networks, all together known as the interactome. Such networks represent functional protein complexes (*e.g*. chaperones), molecular machines (*e.g*. the Sec translocase for protein translocation across the membrane), or highly dynamic cellular pathways (*e.g*. energy transducing systems). High-throughput approaches in yeast and other organisms have revealed that most proteins interact with only few other proteins. In contrast, relatively small numbers of proteins, the so-called "interaction hubs", have multiple interacting partners and thus seem to participate in multiple protein complexes or protein superstructures [52-54]. Furthermore, the available data indicates that flexible protein networks exist in which protein complexes are composed of core proteins and peripheral proteins that readily assemble and disassemble. Thus, the interaction hubs in protein networks can be divided into "date hubs" that are mainly involved in dynamic interactions, and "party hubs" that are involved in permanent interactions. Interestingly, the party hubs often seem to be connected, which suggests that they represent the cores of highly clustered functional modules [52]. It should be noted that interactome studies have so far been predominantly focused on soluble proteins, and only little, if any, data is available for protein networks in membranes.

Interactome studies in *B. subtilis *have mainly involved small-scale protein networks that are related to DNA replication and chromosome dynamics [55,56], cell division [56] and cell morphogenesis [57]. These networks were defined by iterative cycles involving yeast two-hybrid screening ("interactome walking") [55,58]. The data has been deposited in the *B. subtilis *protein interaction database "SPiD" [59,60].

### 1.6 Production of protein complexes

One major challenge for postgenomic research is to produce protein complexes in sufficient amounts for biochemical and structural studies. Several studies have shown the feasibility of purifying endogenous complexes for structure determination, including RNA polymerase II [61] and the ribosome [61,62]. In addition, technical advances such as Tap-tagging have allowed easier purification of large multiprotein complexes [61,63]. This latter approach is currently restricted by the low abundance of many complexes within the cell. However, large scale functional characterization and structure determination of macromolecular complexes requires the purification of the different subunits in large quantities and their assembly into a functional entity. One way to obtain protein complexes from individual proteins *in vitro *involves producing highly purified and soluble proteins at high-concentrations and subsequent formation of protein complexes, which are suitable for further biochemical and crystallographic studies. This technique of *in vitro *reconstitution from separately purified components can be used to study small or mid-size assemblies. The major drawback of this technique is that it is relatively slow and often requires refolding steps. In many cases, proteins that form complexes in cells remain at least partially unfolded in the absence of their normal cellular partners in a heterologous expression system. Frequently, creation of a protein complex from individual proteins is not a simple task and carries along many technical problems. Firstly, over-expression of only one protein from a complex may be the cause for its insolubility [64]. Secondly, posttranslational modifications can not be reproduced during such *in vitro *experiments and further studies may not be successful [65]. In addition, it is often necessary to produce two or more proteins at the same time to obtain proper folding and/or interaction [66]. To overcome some of these difficulties new methods for over-expression of two or more proteins in different hosts have been developed. Co-expression can be achieved by using two or more plasmids each of which bears a gene coding a subunit of a protein complex and a different selection marker. Another way is the introduction of several genes into one expression vector [64]. In bicistronic vectors, despite the presence of ribosome binding sites for each gene, the expression of the second gene is usually much lower. Insertion of a promoter in front of the second gene may improve the yields of the second product [67]. Additionally, construction of a plasmid bearing four genes coding for protein subunits was reported. This method uses LINK sequences and ligation-independent cloning (LIC), which avoids PCR. Thus, the generation of unwanted mutations can be avoided [64].

Efficient production of protein complexes requires suitable purification steps. In comparison with conventional methods like ion-exchange chromatography, size exclusion chromatography or hydrophobic interaction chromatography, affinity tags represent highly efficient tools for complex purification under mild elution condition [68]. The use of different fusion tags can help to identify protein complexes. A clear disadvantage of this method is that the presence of a fusion tag may prevent the interaction with another protein of the complex. Furthermore, mass spectrometry is usually used for identification of proteins in the complex [69].

The new expression systems, from the common binary expression to the more complicated multi-expression systems for production of protein complexes, are well suited for structural proteomics high-throughput strategies as used for the SPINE (Structural Proteomics In Europe) and E-meP (European Membrane Protein consortium) projects. Structural proteomics projects are creating large amounts of data that has to be organized and archived. Recently, the Laboratory Information Management System (LIMS) for structural biology and genomics was developed [70]. In addition, an integrated LIMS system, such as the Protein Information Management System (PIMS) is currently being developed in Europe [71,72]. This system can also handle complicated data, such as information on expression of protein complexes.

Most of the methods and techniques for production of protein complexes that have been mentioned above use as the host *E. coli*,*Saccharomyces cerevisiae*, baculovirus-infected insect cells or mammalian cells. Further development of efficient production systems for protein complexes seems to require the identification of new expression hosts with better characteristics. Clearly, *B. subtilis *is one of such candidate hosts with ample possibilities for improving the level and quality of protein complex production.

## 2. Mechanisms and bottlenecks for membrane protein and protein complex biogenesis

### 2.1 Membrane protein biogenesis

**Figure 3:**
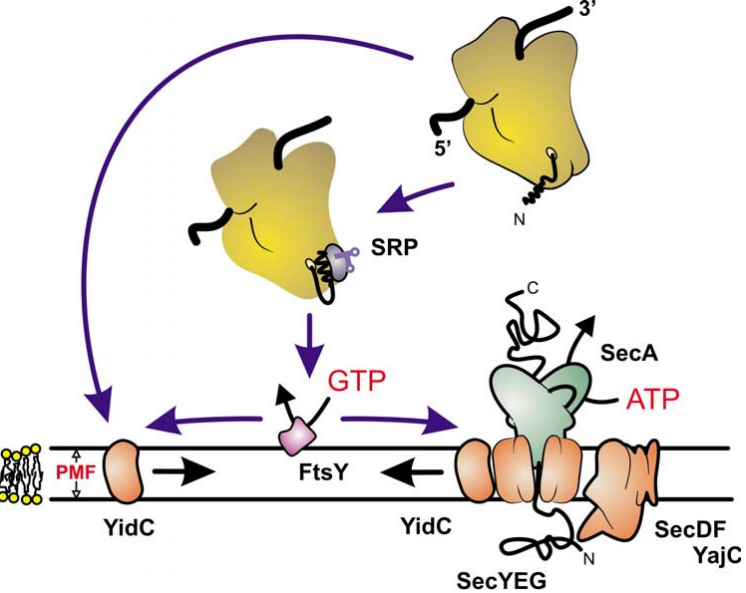
**Scheme of membrane protein targeting and insertion by the Sec translocase and YidC**. The bacterial Sec translocase is a protein complex in the cytoplasmic membrane, which comprises a peripheral motor domain SecA, the protein-conducting channel SecYEG, and the accessory proteins SecDF(yajC) and YidC. Membrane proteins are cotranslationally targeted to the Sec translocase as ribosome-bound nascent chains by the SRP and the SRP-receptor FtsY. FtsY associates with the SecY subunit of the Sec translocase, and associates with SRP in a GTP-dependent fashion. GTP hydrolysis at FtsY and SRP effects the release of the ribosome-nascent chain complex from SRP to the SecY subunit of the Sec translocase. Next, chain elongation at the ribosome is directly coupled to the SecY-mediated insertion of the nascent membrane protein into the cytoplasmic membrane. During membrane insertion, newly synthesized transmembrane segments of nascent membrane proteins contact YidC, which may facilitate the lateral release of these hydrophobic segments into the lipid bilayer and/or assist in their folding and assembly. Translocation of large polar extracellular regions through the SecYEG translocation pore is effected by SecA at the expense of ATP. YidC also acts as a Sec-independent membrane protein insertase for a number of small membrane proteins. These proteins are either targeted directly to YidC, or possibly utilize SRP and FtsY for targeting. How SRP discriminates between SecYEG- and YidC-dependent targeting of nascent membrane proteins is unknown. Abbreviation: PMF, proton motive force.

Membrane protein biogenesis in Gram-positive bacteria like *B. subtilis *is a largely unstudied field of research. The large majority of our knowledge on this process in Gram-positive bacteria is based on bioinformatic studies and comparisons with other organisms, while only a limited number of experimental studies exist. In general, it is believed that membrane protein insertion in Gram-positive organisms follows similar principles as resolved for the Gram-negative *E. coli *(Fig. 3).

#### Targeting to the membrane

In all prokaryotic cells, the biogenesis of proteins starts with translation of the mRNA at the ribosome in the cytoplasm. While cytoplasmic and most secreted proteins are completely translated in the cytoplasm, complete translation of integral membrane proteins poses problems to cells, as these hydrophobic proteins are prone to aggregation and misfolding. Therefore, at an early state once the first transmembrane segment (TMS) or signal peptide emerges from the ribosome, it is bound by a ribozyme, denoted as SRP ("signal recognition particle") [73] in eukaryotes or Ffh ("fifty four homolog") in bacteria. In eukaryotes, this results in a translational arrest, whereas in prokaryotes this phenomenon has not been observed. Subsequently, the SRP (Ffh) – ribosome – nascent chain complex is co-translationally targeted to the membrane, where it binds to the SRP (FtsY) receptor (in *B. subtilis *also denoted as Srb [74]). In *E. coli*, FtsY is bound to the heterotrimeric SecYEG complex, and it has been suggested that a cascade of GTP-binding and hydrolysis events by the heterodimeric Ffh-FtsY complex effect the release of the nascent chain from SRP and the subsequent transfer to the SecYEG translocation channel.

#### Insertion of membrane proteins

Initially two models for membrane protein insertion were postulated: auto-insertion and protein-mediated insertion. The first mechanism proposes the spontaneous insertion of TMS into the lipid bilayer driven by hydrophobic interactions and in some cases directed by the proton motive force (PMF). Evidence for such a mechanism was based mostly on *in vitro *experiments with small membrane proteins such as Pf3 [75] and M13 [76], both coat proteins of bacteriophages, that seemed to insert spontaneously in protein-free liposomes. However, in recent years, it has become clear that these proteins do not insert spontaneously *in vivo *but rather use a pathway that depends on a membrane protein termed YidC. Complex multispanning membrane proteins, however, depend on the general protein translocation pore SecYEG for insertion. Membrane proteins show enormous structural variations in number of TMSs, hydrophobicity of the TMSs, the membrane topology of the TMSs, the length and polarity of the translocated domains and loops, and the oligomeric state of membrane proteins in their functional state.

#### Co-translational membrane insertion via the SecYEG complex

The SecYEG complex consists of three conserved integral membrane proteins SecY, SecE and SecG. The structure of a monomeric SecYEG complex from *Methanococcus jannaschii *has been solved by X-ray crystallography [77], while a low resolution cryo-electronmicroscopy structure of a ribosome-bound dimeric SecYEG complex has been solved from *E. coli *[78]. Currently, there is a controversy about the functional oligomeric state of the SecYEG complex, but experimental evidence demonstrates that in cells, SecYEG complexes assemble as oligomeric, mostly dimeric, entities. The SecYEG complex fulfills a dual function, *i.e*., it both catalyzes the translocation of secretory proteins across the membrane and the membrane insertion of membrane proteins into the lipid bilayer. Secretory proteins are translocated as unfolded polypeptides through an aqueous channel in the SecYEG complex, and this process is driven by ATP binding and hydrolysis by the molecular motor protein SecA that associates with the SecYEG complex (See also below). On the other hand, most membrane proteins insert into the membrane in a co-translocational fashion, which means that while the protein is synthesized at the ribosome, it is concomitantly inserted into the lipid bilayer via the SecYEG complex. Large extracellular loops of membrane proteins, however, need to be translocated completely across the membrane and, depending on their length and polarity, this translocation event requires the activity of SecA. Newly synthesized TMSs are thought to first enter the central pore in the SecYEG complex whereupon they are released into the lipid bilayer via a lateral opening (gate) in the SecYEG complex. Although the subunits of the *B. subtilis *and *E. coli *SecYEG complex exhibit a high sequence similarity [79-81], these proteins do not seem to be functionally exchangeable [82].

A very important subunit of the Sec translocase involved in both protein translocation and membrane insertion is SecA, a cytosolic homodimeric ATPase, which binds to the cytosolic loops of the SecYEG complex. Protein translocation is strictly dependent on SecA, whereas membrane proteins without large extracellular domains insert in a SecA-independent manner. SecA is however needed to drive the translocation of extracellular polar domains of membrane proteins. The ATPase activity of SecA is highly stimulated by the presence of membranes, SecYEG and a translocation-competent precursor protein [83]. *B. subtilis *and *E. coli *SecA are only partially exchangable in functional terms, suggesting some degree of species specificity. In general, the degree of functional exchangeability within the Gram-positives is higher than between Gram-positive and -negative bacteria [84,85]. Interestingly, some Gram-positive bacteria such as *Bacillus anthracis*, *Corynebacterium diphtheriae*, *Listeria monocytogenes*, *Staphylococcus aureus*, *Staphylococcus epidermidis*, *Streptococcus gordonii*, *Streptococcus parasanguis*, and mycobacteria, contain two paralogous SecA proteins. One of these paralogues (SecA1) is involved in the general housekeeping functions of protein translocation, whereas the other paralogue (SecA2) is required for the secretion of a subset of secretory proteins only. SecA2 proteins have sofar not been implicated in membrane protein insertion [86].

SecDFYajC is another heterodimeric membrane protein complex that was found to associate with the SecYEG channel in *E. coli *and that is needed for efficient protein translocation *in vivo*. Homologues of all three proteins were identified in *B. subtilis*, but these differ in two aspects from the equivalent *E. coli *proteins. Firstly, SecDF of *B. subtilis *is a single polypeptide and secondly, the *yrbF *gene, which encodes for the YajC homologue of *B. subtilis*, is located in a locus separate from *secDF *[87].

#### YidC mediated membrane protein insertion

Proteins homologous to the Alb3/Oxa1/YidC superfamily are found in all domains of life and were shown to facilitate the insertion of some membrane proteins independently of the SecYEG complex. Oxa1 and Alb3 are proteins of the inner membrane of mitochondria and the thylakoidal membrane of chloroplasts, respectively. The *E. coli *YidC is the best described member of this protein family and functions as a membrane protein insertase for a specific subset of proteins. YidC is involved in the membrane insertion of some of the subunits of the major energy transducing complexes in the cytoplasmic membrane, and it catalyzes the membrane insertion of the small bacteriophage coat proteins that were previously thought to insert spontaneously. YidC can either function on its own, or co-operate with the SecYEG complex to facilitate membrane protein insertion. The membrane insertion of subunit c of the *E. coli *ATP synthase solely requires YidC [88], whereas CyoA, a subunit of the cytochrome o oxidase, requires both YidC and SecYEG [89]. Some Gram-positive bacteria, such as *B. subtilis*, contain two paralogues of the YidC protein. In *B. subtilis*, these YidC paralogues are known as SpoIIIJ and YqjG [90]. The exact role of these two proteins in membrane protein insertion is unknown although SpoIIIJ seems to fulfill a specific function in sporulation. However, gene inactivation studies have shown that the presence of only one of the two proteins (SpoIIIJ or YqjG) is essential for viability. Previous studies suggest that these proteins impact on post-translocational stages in protein secretion rather than membrane protein insertion, although from the conserved function of Oxa1/Alb3/YidC family a function in membrane protein insertion is expected [90]. Importantly, these membrane protein insertases may also function at the post-insertional stage. YidC may facilitate the proper folding of the newly inserted membrane protein and stabilize these proteins prior to their assembly into oligomeric membrane protein complexes as suggested for the formation of the ring-like F_0_-sector of the F_1_F_0_-ATPase[88].

### 2.2 Protein complex biogenesis – the bacterial divisome

In *B. subtilis*, as well as in all living cells, genes involved in a given cell function are activated at the time of execution of that function. Also, the genes encoding proteins that function in complexes are co-expressed, and temporal cascades of gene expression control multiprotein structure biogenesis. These multiprotein structures have a crucial role to direct complex processes during the cell life cycle. Studies on the formation of these protein superstructures require the most advanced technologies of molecular biology. In general, these multiprotein structures are built from more or less stable proteins and sub-complexes and some of them are amenable to purification, typically by affinity methods, and to subunit identification by mass spectrometry. One of the most extensively studied protein structures in *B. subtilis *is the divisome, a structure that is composed of division proteins and proteins involved in their biogenesis. Therefore, the divisome serves an important model function for studies on *B. subtilis *as a producer of protein complexes.

**Figure 4:**
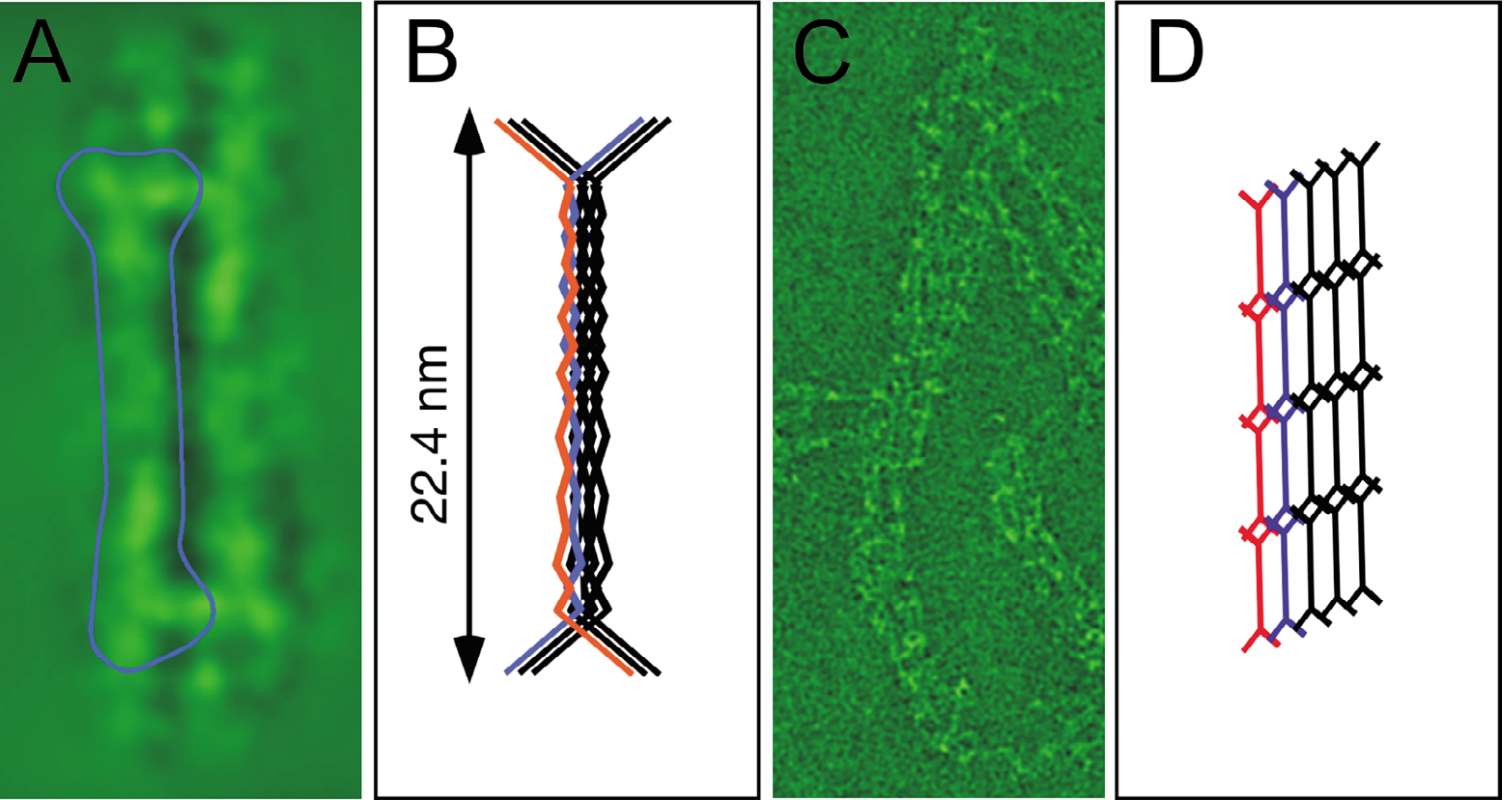
**DivIVA oligomers form a two-dimensional network as judged from cryonegative stain transmission electron microscopy images**. **A) **Freshly purified DivIVA appears as a "doggy-bone" shaped particle. **B) **A tentative model for the hexameric DivIVA oligomer. **C) **Further oligomerization of DivIVA "doggy-bones" leads to two-dimensional network formation. **D) **A tentative model for the two-dimensional DivIVA network [100].

**Figure 5:**
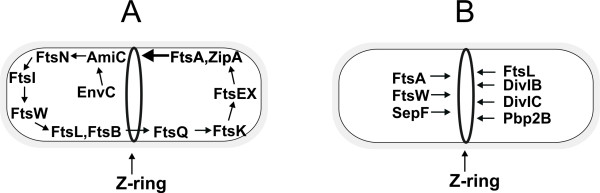
**Formation of the divisome protein complex in *E. coli *and in *B. subtilis***. **A) **Model for the assembly of proteins into the septal ring of *E. coli*. First, FtsZ forms a Z-ring. FtsA and ZipA are recruited next, independently from one another. Once both FtsA and ZipA have localized, the remaining proteins join the ring in the order indicated. **B) **Model for assembly of proteins into the septal ring during vegetative growth of *B. subtilis*. The assembly of late vegetative division proteins (FtsL, DivIB, DivIC and Pbp2B) is not linear and these components appear to assemble in a completely interdependent manner.

Cell division in bacteria is a complex process involving the coordinated participation of a group of proteins which assemble at the division site into a multiprotein complex called the divisome (for reviews see [91-94]). This process has been best studied in two bacterial model systems: *E. coli *and *B. subtilis*. The earliest apparent event in cell division is the formation of an FtsZ ring (Z-ring) at the future septum site. In *B. subtilis*, the MinC and MinD proteins form a complex which blocks the formation of the Z-ring at the cell poles, whereas the nucleoid blocks the septation at mid-cell. The topological control of MinCD activity is provided by DivIVA in *B. subtilis *[95,96] and by oscillating MinE in *E. coli *[97-99]. DivIVA can form oligomers which serve as building blocks in the formation of higher order assemblies giving rise to two-dimensional lattices in a time-dependent manner (see Fig. 4) [100]. DivIVA is stably associated with the cell poles, to which it recruits MinCD, probably by direct interaction with MinD [101,102]. The initiation of septation is a complex process involving many proteins and their complexes as well as specific cell cycle conditions, such as DNA replication and segregation. The protein complexes respectively involved in these processes are known as the replisome and segresome. The FtsZ protein assembles into a cytokinetic ring on the inner surface of the cytoplasmic membrane at the place where division will occur [103,104]. The Z-ring structure provides the framework for the recruitment or assembly of about ten membrane and cytoplasmic proteins, uniquely required for cell division. Some of these are required for biogenesis of the new hemispherical poles of the two daughter cells. In *E. coli*, during cell division the proteins assemble in a defined order as follows: FtsZ, FtsA/ZipA, (FtsE, FtsX), FtsK, FtsQ, (FtsB, FtsL), FtsW, FtsI, FtsN, AmiC and EnvC, where the proteins in parentheses assemble simultaneously (Fig. 5A) [105]. The assembly of the Z-ring depends on FtsA and/or ZipA, while the localization of the latter pair of proteins depends on FtsZ [106,107]. FtsK does not require any downstream proteins to assemble at the Z-ring. FtsL and FtsB localize in a co-dependent fashion [108]. The localization of the last protein from this set, EnvC, depends on all of the other proteins. This hierarchical localization of division proteins in *E. coli *is likely to reflect a sequence of protein-protein interactions that lead to the assembly of the protein complexes of the divisome. *B. subtilis *has homologues of most of the *E. coli *division proteins, including FtsZ, FtsA, ZipA (possible functional homologue of *B. subtilis *EzrA), FtsL, YgbQ (DivIC in *B.subtilis*), FtsQ (DivIB in *B. subtilis*), FtsW (YlaO in *B. subtilis*), and Pbp3 (Pbp2B in *B.subtilis*) (reviewed in [91]). However, SepF is only present in *B. subtilis *[109]. In contrast to the hierarchical localization of division proteins in *E. coli*, in *B. subtilis *the equivalent division proteins are recruited in a more concerted manner (Fig. 5B) (reviewed in [91]). DivIB, DivIC, FtsL, Pbp2B and probably YlaO are all completely interdependent in their assembly at the division site and depletion of FtsA, DivIC, FtsL or Pbp2B, abolishes the positioning of the other cell division proteins at mid-cell. The first three proteins from this division protein set, DivIC, FtsL and DivIB, seem to form one or more different oligomers [110]. The possible role of FtsL is to stabilize DivIC through formation of a DivIC-FtsL complex [111] and DivIB has a role in FtsL turnover [112]. The function of YlaO is closely connected to FtsL and likely to include targeting of cognate PBPs (penicillin binding proteins).

The dynamics of division protein complexes have been interrogated by mapping and mutation analysis. Many of these physical interaction studies have been complemented by genetic and phenotypic screens [91]. Microtubule and cytoskeletal superstructures have also been subject to proteomic analyses. Genome-wide datasets and ever-more-complex networks built from such data seem to quickly overload human intuitive capacity. Inevitably, these vast amounts of information must be captured and processed in mathematical models, as has been done in the physical sciences for many years. User-friendly interfaces for simulation of biological systems have been created that will certainly be widely used to explore the manifestations of biochemical and genetic networks. The cell cycle has in fact long been subject to modelling efforts, which have become increasingly sophisticated and coupled to experimental tests of model predictions. A good example of intensive modelling is the oscillation of the MinCDE complex in *E. coli *which has been analysed using simple reaction-diffusion mathematical models [113-115] as well as more advanced mathematical models [116,117].

The dynamics of division protein complexes have been interrogated also by mapping and mutation analyses. It is clear that a complete appreciation of the mechanism of divisome assembly in *B. subtilis *will require a much deeper understanding of the protein-protein interactions that take place between divisome proteins both before localization at the division site and during assembly of the divisome. Many of these physical interaction studies have been complemented by genetic and phenotypic screens [91]. Some of the septasomal proteins were expressed, purified and used for biochemical and crystallographic studies (for reviews see [89,92]). Although a huge amount of data about the divisome exists, development of an efficient *B. subtilis *production system for protein complexes will be required for the *in vitro *reconstitution of this and other crucial cell cycle protein complexes. In turn, the knowledge thus generated is likely to lead to important insights and tools for removal of bottlenecks in the production of protein complexes in *B. subtilis*.

### 2.3 Molecular chaperones

Molecular chaperones are cellular components, which assist folding processes of proteins by interacting with non-native polypeptide chains in a non-covalent manner. This definition excludes classical enzymes involved in catalyzing protein folding with covalent reactions, although in some folding factors these both types of activities can be distinguished; there may be an enzyme domain and a chaperone domain in the same component. Chaperones are typically cytosolic proteins or multiprotein complexes involved in protein folding assistance in various cellular processes. The roles of microbial molecular chaperones in protein folding assistance, aggregation prevention, protein quality control, chaperone-assisted protein degradation and the heat shock response have been extensively studied with cytosolic and secreted protein substrates and proteomes [118], but in the context of membrane protein folding and assembly, information on such functions of chaperones is scarce. Although most of the mechanistic studies of bacterial chaperones have been carried out with *E. coli*, the available information can also be applied to other bacterial systems including *B. subtilis*. When proteins are overexpressed in bacteria regardless of whether they are cytosolic, secreted or membrane proteins, a general problem is the formation of insoluble aggregates of predominantly misfolded proteins, so called inclusion bodies. Co-overexpression of chaperones may help in controlling the folding process of overexpressed proteins and thereby decrease aggregation.

#### General cytoplasmic chaperones

Two major cytosolic chaperones with a general role in bacterial cytoplasmic protein folding are GroEL/GroES and DnaK/DnaJ. The GroEL chaperonin and its co-factor GroES form a ring-shaped ATP-dependent protein folding machine and a "folding-friendly" environment in the GroEL/GroES cavity for newly translated proteins. Proteomic studies have identified in *B. subtilis *28 potential GroEL/GroES substrates, all cytosolic proteins [119], and in *E. coli *the GroE-dependent proteins account for about 10% of all cytoplasmic proteins [120]. The bacterial signal recognition-like particle (SRP) is a chaperone-like component involved in co-translational targeting of extracytoplasmic proteins, including membrane proteins, to the Sec translocase [121-124]. Since the co-translational targeting process is strictly controlled by SRP and SRP-bound nascent polypeptides can stay insertion competent for long times, it may be that general intracellular molecular chaperones are needed in limited extent for the folding and aggregation prevention of extracytoplasmic proteins in normal conditions. However, when misfolded and aggregated secretory or membrane proteins are formed in the cytosol under stress conditions, and when proteins are overexpressed, chaperones are found associated with the protein aggregates. This is consistent with the general property of these folding factors to interact with non-native polypeptide chains. Overexpression of membrane proteins fused to green fluorescence protein in *E. coli *resulted in accumulation of cytosolic multiprotein aggregates consisting of the produced protein, GroEL/GroES and DnaK/DnaJ chaperones, cytoplasmic proteases as well as precursors of several periplasmic and outer membrane proteins [125]. On the other hand, it has been shown that GroEL forms *in vitro *a soluble complex with bacteriorhodopsin (BR) and the complex-bound BR folds in the presence of ATP to its functional native conformation. This folded BR can be transferred efficiently to liposomes [126,127]. In a similar GroEL-dependent manner the phage lambda holin is delivered to liposomes [128]. These results suggest that GroEL/GroES may affect membrane protein assembly in bacterial cells. The DnaK chaperone, its co-chaperone DnaJ and the trigger factor, a ribosome-bound protein with a dual chaperone and peptidyl-prolyl *cis*-*trans *isomerase activity, have overlapping functions in the folding of nascent polypeptides [129]. Effects of co-overexpression of these general chaperones on inclusion body formation and membrane insertion of the overexpressed CorA magnesium transporter in *E. coli *have been studied [130]. An interesting finding was that CorA inclusion body formation was prevented by co-overexpression of DnaK/DnaJ [130]. CorA was also inserted into the cytoplasmic membrane more efficiently in DnaK/DnaJ overexpressing cells. In contrast, co-expression of GroEL/GroES, SRP or the translocation ATPase SecA had little or no effect on CorA inclusion body formation. In *B. subtilis *these chaperones are required for heat shock survival [131], but their significance for the folding of nascent polypeptides and aggregation prevention is still unclear. However, it has been demonstrated that overexpression of both GroEL/GroES and DnaK chaperone systems in the *hrc *repressor null mutant of *B. subtilis *improves secretion of a single chain antibody fragment and decreases inclusion body formation in the cytosol [24]. This suggests that co-overexpression of molecular chaperones decreases aggregation of heterologous proteins and increases their yields also in *B. subtilis *expression systems. Further studies are needed to find out whether chaperone co-expression can enhance yields of membrane proteins expressed in *B. subtilis*.

#### Dedicated chaperones

In addition to the general molecular chaperones, bacteria contain many other chaperones with more dedicated roles in protein folding. CsaA is a secretion-related chaperone-like protein of *B. subtilis*, which suppresses the growth defects of *E. coli *mutants of the major chaperones, interacts with the SecA translocation ATPase and stimulates translocation of prePhoA into *E. coli *membrane vesicles bearing the *B. subtilis *translocase [132-134]. It is not known whether CsaA has any role in the targeting and chaperoning of membrane proteins. The *B. subtilis *ClpX, a chaperone belonging to the AAA+ superfamily of ATPases, modulates the assembly of the tubulin-like protein FtsZ independently of its protease partner ClpP and ATP hydrolysis and thereby regulates the formation of the Z-ring and cell division [135]. ClpX inhibits FtsZ polymerization, increases the pool of soluble FtsZ in the cell and affects the dynamics of the cell septum formation. There is also evidence that some proteases involved in protein quality control are chaperones. The membrane-bound HtrA and FtsH are examples of proteases having chaperone-like properties [136-138]. The formation of correct protein structures is often not only dependent on the proper chaperones but additionally various foldase enzymes assist folding processes both in the cytosol and the periplasmic space. As an example, in *B. subtilis*, the peptidyl-prolyl *cis*-*trans *isomerase PrsA affects the posttranslocational folding and stability of proteins at the membrane-cell wall interface [25,139-143].

### 2.4 Protein quality control and protein turnover

*B. subtilis *has an extensive quality control system for protein production. This system can respond to the presence of misfolded or incompletely synthesized proteins by activating proteases that remove these proteins. The biotechnological advantage of the quality control system is that it enables the production of high quality proteins with few impurities of misfolded side-products. However, it can also represent one of the major bottlenecks for the production of especially heterologous proteins. Besides their role in protein quality control, proteases are also involved in the removal of cleaved signal peptides and in the processing of precursor proteins in order to acquire the active mature forms of these proteins. Furthermore, proteases are highly important for many regulatory processes within the cell.

#### Cytoplasmic protein quality control

Nascent proteins often expose strands of amino acids that are susceptible for degradation or aggregation. Usually cytoplasmic proteins fold rapidly, thereby hiding the susceptible parts of the protein from their surface and rendering the protein intrinsically stable and resistant against degradation. Many proteins do not fold rapidly enough by themselves, and their folding is catalyzed by chaperones. Also, under conditions where the presence of misfolded or unfolded proteins is induced (for example by heat-shock, overproduction of proteins, or production of heterologous proteins) the chaperones can enhance the folding process and thereby prevent the accumulation of these proteins in the cytoplasm.

**Figure 6:**
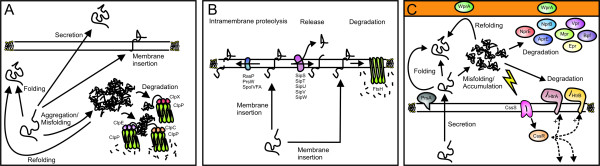
**Protein quality control and proteolysis**. **A) **Model for cytoplasmic protein quality control in *B. subtilis *to which cytoplasmic proteins, membrane proteins and secretory proteins are subject. Depending on the presence or absence of targeting signals, newly synthesized proteins can be targeted for secretion or membrane insertion, or they can remain in the cytoplasm. If control of their folding by chaperones is insufficient these proteins can misfold and/or aggregate. This can lead to degradation by proteases such as ClpCP, ClpEP or ClpXP. Alternatively, misfolded proteins can be refolded with the help of chaperones. **B) **Model for protein quality control and degradation of membrane proteins within the membrane of *B. subtilis*. Proteins targeted to the membrane can be subject to processing by signal peptidases (*e.g*. SipS-W) or to degradation by membrane-associated proteases such as FtsH, PrsW, RasP or SpoIVFA. **C) **Model for extracytoplasmic protein quality control and degradation in *B. subtilis*. Translocated secretory proteins can fold with the help of folding catalysts such as PrsA. Accumulation of misfolded translocated proteins at the membrane-cell wall interface can trigger a secretion stress response, involving the CssRS two-component regulatory system. If activated, CssRS causes the up regulation of membrane-associated proteases such as HtrA and HtrB. These two proteins can probably catalyze both protein degradation and protein folding. Misfolded proteins are furthermore subject to degradation by cell wall-associated and/or secreted proteases, such as AprE, Bpf, Epr, Mpr, NprB, NprE, Vpr and/or WprA.

The function of the cytoplasmic protein quality control system is to remove the proteins from the cytoplasm that are not folded correctly (Fig. 6A). In addition to malfolded proteins, this system eliminates "unemployed" proteins, which are no longer integrated into functional complexes and thereby are no longer protected against proteolytic attack [144]. In both prokaryotic and eukaryotic organisms the Clp proteases (caseinolytic proteases) appear to play pivotal roles in cytoplasmic protein quality control [145-147]. The Clp proteases generally function as complex molecules. These consist of Clp ATPase subunits forming hexameric rings that are attached to two central heptameric rings of ClpP subunits. Thereby, the ClpP subunits form a central proteolytic chamber [148,149]. It seems that the entrance to the proteolytic chamber is too small for folded proteins to enter. Accordingly, it is generally believed that misfolded proteins are first unfolded by the Clp ATPases and, subsequently, transferred to the central proteolytic chamber. There, they are degraded by the ClpP protease subunits. The exact mechanism of entry and exit of proteins and peptides into and from the ClpP chamber is a subject of ongoing study.

In *B. subtilis *it appears that the ClpP peptidase indeed is a key component in the protein quality control system, and a knockout of *clpP *renders *B. subtilis *highly susceptible for protein aggregation [150]. Accordingly, *clpP *mutants do not grow at high temperatures [151]. Due to the degradation of several regulators of important cellular processes by ClpP, mutations in *clpP *result in a pleiotropic phenotype, which includes loss of motility, competence development and sporulation [151]. There are three distinct Clp ATPases known in *B. subtilis *(ClpC, ClpX and ClpE), which are all able to form functional complexes with ClpP [152-154]. ClpCP, ClpEP and ClpXP each appear to have different substrate specificities. Moreover, these substrate specificities can additionally be modified by adaptor proteins [155], which are used by the cell to target ClpP degradative activity towards specific proteins. By this mechanism, the action of ClpP in processes, like competence development and sporulation, is modulated.

The Clp proteases belong to the class III heat shock proteins. All genes encoding for this class of proteins, except *clpX*, are regulated by the repressor CtsR. CtsR is present at a basal steady-state level in the cells, and several proteins can influence its degradation (e.g. ClpCP, ClpEP) or modify its activity (e.g. McsA, McsB) [156]. Via these mechanisms the activity of Clp proteases is up regulated when misfolded proteins or protein aggregates start to accumulate.

In addition to the Clp proteases, also other proteases are present in the cytoplasm, such as HlsUV, Lon and the membrane-associated FtsH protein. These proteases, which also depend on ATPase activity, belong to the same superfamily of proteins, the AAA+ superfamily. Many more (putative) proteases and peptidases, not belonging to the AAA+ superfamily, are present in the cytoplasm of *B. subtilis*, including several metalloproteases. They however do not seem to be very important for general cytoplasmic protein quality control in *B. subtilis*.

#### Integral membrane protein turnover and quality control

The first evidence of membrane protein degradation in *B. subtilis *has come from proteomic studies, which showed the presence of predicted membrane proteins in the culture medium [157,158]. For some of these proteins it has been shown that their release from the membrane probably depends on cleavage by type I signal peptidases (SPases; Fig. 6B). For most of these proteins, however, it is still unknown which proteases are responsible for their release [157]. Type I SPases are proteases that remove the signal peptides from secretory proteins after their translocation from the cytoplasm to the extracellular environment. Five type I SPases are present in *B. subtilis*: SipSTUVW, of which SipS and SipT are of major importance for protein secretion and SipU, SipV and SipW only seem to have minor roles [159,160]. The type I SPases are membrane proteins with their active site located at the extracytoplasmic side of the membrane. The consensus sequences for type I cleavage in *B. subtilis *are well defined [13]. However, in several of the proteins that were found in the medium such a cleavage site was absent, and the deletion of SPase-encoding genes did not affect the presence of most of these proteins in the medium [157].

Several examples are currently known of cleavage of membrane proteins within the membrane itself. Most of these concern Regulated Intramembrane Proteolysis (RIP; Fig. 6B). In this process, a membrane protein is cleaved in order to release the cytoplasmic part as well as the proteins interacting with this cytoplasmic part, which can subsequently engage in processes, such as gene transcription. An example of this process is the cleavage of RsiW, an anti-sigma factor that modulates the activity of σ^W^. RsiW appears to be cleaved in two steps by PrsW (site-1-proteolysis) and RasP (site-2-proteolysis) in order to release σ^W ^[161,162]. Another example of RIP in *B. subtilis *concerns the maturation of the sigma factor σ^K^: pro-σ^K ^is activated through site-2-proteolysis by the membrane protease SpoIVFB. To start this process, the SpoIVFB itself is activated by site-1 proteolysis of SpoIVFA, the repressor of SpoIVFB. Site-1 proteolysis of SpoIVFA can be catalyzed either by SpoIVB or by the CtpB protease [163-165].

Protein quality control of membrane proteins involves different stages. Mistargeted or misassembled integral membrane proteins likely already aggregate in the cytoplasm due to their high hydrophobicity. Therefore, quality control of integral membrane protein insertion may at least partially occur via cytoplasmic protein quality control mechanisms. Within the membrane at least one mechanism of quality control for integral membrane proteins is known to exist [166]. This involves the proteolytic activity of FtsH, a membrane-anchored member of the AAA+ superfamily. The proteolytic domain of FtsH is exposed in the cytoplasm. The known substrates of FtsH include both short-lived regulatory proteins in the cytosol and unassembled subunits of membrane protein complexes in the membrane [166]. FtsH has been shown to degrade SecY when not assembled in a stable complex with secE [167]. Also, the subunit *a *of the proton ATPase F_0 _sector [168,169] and the protein of unknown function YccA [170] are membrane proteins that become degraded by FtsH when not properly assembled. A remarkable property of FtsH is its ability to dislocate substrate proteins from the membrane to allow their degradation [166].

FtsH, like other AAA+ family members, forms homohexameric complexes [170,170,172]. It has been shown that the FtsH homohexamer of *E. coli *forms a complex with membrane embedded HflKC complexes. The entire supercomplex of FtsH and HflKC is also known as the FtsH holoenzyme [171,173,174]. The function of HflKC is thought to be inhibition of FtsH-mediated proteolysis of membrane proteins, thereby increasing the capacity to degrade soluble substrates. Interestingly, YccA, a protein that itself is degraded by FtsH, is another modulator of FtsH proteolytic activity towards integrated membrane proteins [170]. Notably, HflKC seem to be absent from *B. subtilis*. In contrast, a protein with a low level of sequence similarity to *E. coli *YccA is encoded by the *B. subtilis *genome. In the absence of functional data, it is presently unclear how the activity of FtsH is modulated in this organism.

In addition to FtsH, the membrane-bound metallo protease HtpX has been implicated in the quality control of *E. coli *membrane proteins, like SecY [175]. Interestingly, a homologue of HtpX, known as YkrL, is present in *B. subtilis*. The presumed role of YkrL in protein quality control awaits detailed investigations.

#### Extracytoplasmic quality control and secretion stress

*B. subtilis *secretes high amounts of proteases into its medium, which degrade proteins that do not fold properly or that fold slowly (Fig. 6C). The importance of the presence of these extracellular proteases in relation to (industrial) protein production is illustrated by the application of the WB800 strain, which lacks 8 extracellular proteases [21]. Practically all extracellular proteolytic activity is abolished in this strain. The use of the WB800 strain has enabled the production of various heterologous proteins, which normally are rapidly degraded after secretion. Interestingly, even the production of homologous proteins can be boosted by removal of these proteases.

Secretion stress occurs when misfolded and/or aggregated proteins accumulate at the membrane-cell wall interface (Fig. 6C). This can be caused for example by overproduction of secretory proteins, or by depletion or inactivation of PrsA. The two-component system CssRS (Control of secretion stress regulator and sensor) plays a pivotal role in the response to secretion stress, as it responds to the accumulation of misfolded proteins at the membrane-cell wall interface [16,17,176]. Upon stimulation of CssRS several proteins are up regulated, including the proteases HtrA and HtrB [16]. HtrA and HtrB are negatively auto- and cross regulated and can substitute at least partially for each others activity [177]. HtrA and HtrB are both membrane-bound serine proteases with their active site located at the extracellular side of the membrane. Notably, HtrA has also been detected in the medium of the cells due to cleavage of the transmembrane segment, whereas HtrB is not detected in the medium [136]. Whether there is a functional role for HtrA in the medium remains to be determined. A double knockout of *htrA *and *htrB *causes up regulation of transcription of *cssR *and *cssS *and results in growth defects and temperature sensitivity [16,177], indicating that both proteases have important roles in combating the detrimental effects of heat. Because the active sites of membrane-associated HtrA and HtrB are located very close to the membrane, it is possible that HtrA and HtrB can cleave the extracellular domains of integral membrane proteins. However, until now there is no documented evidence of such events. In addition to the transcriptional up regulation of *htrA *and *htrB *also other genes are up regulated in a response to secretion stress. These include genes for a putative Mg^2+^-transporter (*yqxL*), several cytoplasmic chaperones and the *liaIHGFSR *operon [178]. The latter operon also seems to be involved in the response to cell envelope stress induced by several antibiotics [179].

Finally, a protein that seems to be involved in extracytoplasmic protein quality control is WprA, a cell-wall bound protease. Notably, WprA is processed into two cell wall proteins: CWBP52, with a serine protease activity domain, and CWBP23, which may have chaperone-like activity [180,181]. Although the WprA processing products are cell-wall bound, they are also found in the culture medium [182]. Production of α-amylase from *B. licheniformis *by *B. subtilis *is enhanced in a knockout of *wprA *[183]. Altogether, it is thought that the WprA CWBP52 product degrades various secretory proteins before they are released into the medium. By contrast, the CWBP23 product may assist in folding of several cell wall-bound proteins [183]. WprA has been shown to be responsible for the degradation of at least one membrane protein: a site-specific mutant of SipS (D146A) [184]. The importance of WprA for the stability of other membrane proteins remains to be determined.

## 3. Conclusions – Perspectives for production of membrane proteins and protein complexes in *B. subtilis*

As outlined in this review, *Bacillus subtilis *is capable of producing and secreting large amounts of high quality proteins. Much is already known about the mechanisms that affect the biogenesis, membrane translocation and stability of these proteins. In contrast, our current understanding of the biogenesis of membrane proteins and protein complexes in *B. subtilis *is still relatively limited. Nevertheless, the high potential of *B. subtilis *for protein production gives confidence that this versatile host organism can also be exploited for producing protein complexes and membrane proteins in order to facilitate their functional and structural analysis. Future research towards achieving these goals needs to focus on the identification and modulation of those quality control systems that are counter-productive with respect to the production of high quality protein complexes and membrane proteins, and on enhancement of the activity of those systems that facilitate the assembly of these proteins. This will require the characterization and engineering of (1) the cellular machinery required for the assembly of cytoplasmic protein complexes and membrane proteins, and (2) the relevant quality control mechanisms in the cytoplasm and membrane that govern protein degradation. Such research will most likely result in the development of entirely new protein production strategies. We consider this feasible, because previous research has successfully identified key bottlenecks in the secretory pathway of *Bacillus*, and has demonstrated that different proteins are affected by these bottlenecks to very different extents. In many cases, this concerned components of quality control systems [17-20,185,186]. Major available resources to further enhance the *Bacillus *cell factory include a detailed knowledge about all essential genes of *B. subtilis*, as well as a collection of more than 3000 mutant *B. subtilis *strains [9]. These mutants can be used to monitor the functionality of expressed proteins from *B. subtilis *and other Gram-positive bacteria through complementation. Importantly, the mutant collection includes strains that lack one or more cytoplasmic, membrane-associated or secreted proteases. The latter strains can be employed to prevent product degradation. Other available resources include previously developed strains, vectors, tools and techniques for a rapid and accurate identification of the specific production bottlenecks of cytoplasmic protein complexes and membrane proteins that are currently either recovered in low quality (*e.g*. mis-translated, aggregated, misfolded, degraded) and/or at low concentrations. With the exception of *E. coli*, such combined resources are presently not available for other bacterial expression systems, such as *Lactococcus lactis*. In conclusion, *B. subtilis *seems perfectly placed for future application as an expression system for the production of protein complexes and membrane proteins, especially those derived from Gram-positive bacteria and pathogens. Research in this direction will certainly result in technical strategies to overcome current bottlenecks, and lead to the development of super-producing strains.

## Competing interests

The author(s) declare that they have no competing interests.

## Authors' contributions

JCZ and JMvD wrote "History", "*B. subtilis *as a host for protein production", "Production of membrane proteins", "Protein quality control and protein turnover", and "Perspectives for production of membrane proteins and protein complexes in *B. subtilis*". DB, MH and JMvD wrote "The membrane as a resource for biomedically and biotechnically relevant proteins". IB, LV and JMvD wrote "Protein complexes and the interactome", "Production of protein complexes and Protein complex biogenesis – the bacterial divisome". AJMD and MJ wrote "Membrane protein biogenesis". VPK wrote "Molecular chaperones". Compilation and final editing of the chapters was done by JMvD, AJMD and JCZ. All authors have read and approved the final manuscript.
